# Using a pupal exuvia to designate the undamaged neotype of a species belonging to a complex of sibling species – the case of *Aedes coluzzii* (Diptera, Culicidae)

**DOI:** 10.1051/parasite/2022020

**Published:** 2022-03-29

**Authors:** Audric Berger, Gilbert Le Goff, Philippe Boussès, Nil Rahola, Jean-Baptiste Ferré, Diego Ayala, Vincent Robert

**Affiliations:** 1 MIVEGEC, Univ. Montpellier, CNRS, IRD 911 avenue Agropolis BP 64501 34394 Montpellier cedex 5 France; 2 EID Méditerranée 165 avenue Paul-Rimbaud 34184 Montpellier Cedex 4 France; 3 Unité d’Entomologie Médicale, Institut Pasteur de Madagascar BP 1274 Avaradoha Antananarivo 101 Madagascar

**Keywords:** Mosquito, Neotype, Molecular identification, Species complex, Aedini, *Aedes detritus*

## Abstract

The mosquito species *Aedes* (*Ochlerotatus*) *coluzzii* Rioux, Guilvard & Pasteur, 1998 was distinguished from its sibling species *Aedes detritus* (Haliday, 1833) using an isoenzymatic method that required the destruction of the entire specimen, therefore no holotype was designated by the species authors. We aimed to designate a neotype for *Ae. coluzzii* from specimens collected from the type-locality and individually reared up to adult stage. Genomic DNA was extracted from pupal exuvia and ITS2 was sequenced, enabling verification of the identity of each specimen as *Ae. coluzzii* or *Ae. detritus*. Among the series of *Ae. coluzzii*, a male was designated as neotype and deposited in a collection. To our knowledge, this is the first time the type of a mosquito species is deposited thanks to its molecular identification from its pupal exuvia. The set of identified specimens allowed additional phylogenetic and morphologic studies.

## Abstract (fr)

L’espèce de moustique *Aedes (Ochlerotatus) coluzzii* Rioux, Guilvard & Pasteur, 1998 a été distinguée de son espèce jumelle *Aedes detritus* (Haliday, 1833) par une méthode isoenzymatique qui a nécessité la destruction de l’ensemble du spécimen, et donc aucun holotype n’a été désigné par les auteurs de l’espèce. Notre objectif était de désigner un néotype pour *Ae. coluzzii* à partir de spécimens collectés dans la localité-type et élevés individuellement jusqu’au stade adulte. L’ADN génomique a été extrait de l’exuvie nymphale et l’ITS2 a été séquencé, permettant la vérification de l’identité de chaque spécimen comme *Ae. coluzzii* ou *Ae. détritus*. Parmi la série d’*Ae. coluzzii*, un mâle a été désigné comme néotype et déposé dans une collection. À notre connaissance, c’est la première fois que le type d’une espèce de moustique est déposé grâce à l’identification moléculaire à partir de son exuvie nymphale. L’ensemble des spécimens identifiés a permis des études phylogénétiques et morphologiques complémentaires.

## Introduction

The bulk of insect taxonomy remains grounded on morphological features. Notable exceptions reside in some groups of insects nowadays investigated intensively, where taxonomy is based on evolutionary notions, and most notably on genetic features that generated the concepts of species complex and sibling species [11].

When describing a new species, the designation of a single physical example of an organism is mandated by the International Code of Zoological Nomenclature (ICZN) to validate the species name. This single organism becomes the name-bearing type for the nominal species. It is defined in the ICZN as the holotype [6]. The designation of a holotype is easy in the framework of the morphospecies concept, but it may become difficult for complexes of morphologically identical species. In this context, the case of the Detritus Complex, composed of two species that cause heavy nuisance, is informative.

On the one hand, *Aedes* (*Ochlerotatus*) *detritus* (Haliday, 1833) was described from specimens collected at Holywood, Downshire, England by means of morphological criteria [5]. On the other, *Aedes* (*Ochlerotatus*) *coluzzii* Rioux, Guilvard & Pasteur, 1998 was described from specimens collected at Salin-de-Giraud, Bouches-du-Rhône, France, using an iso-enzymatic profile [12, 13]. The two species do not present morphological characters that can be employed to distinguish both sibling species grouped in the Detritus Complex [12]. *Aedes detritus* is widely distributed in the Western Palearctic up to Mongolia and *Ae. coluzzii* is restricted to the Western Mediterranean (see [14] for more information and references related to geographic distribution). They exhibit various preferences in their bio-ecology. *Aedes detritus* tolerates a large range of salinity for breeding places, is anautogenous (requires blood-feeding to maturate progeny) and eurygamous (needs large space for mating). *Aedes coluzzii* prefers hypersaline environments such as salt marshes, is possibly autogenous in autumn, and stenogamous (able to mate in a confined volume) (references in [2]). During the 20th century, no reliable and straightforward feature existed to indisputably differentiate the two species, except the historical iso-enzymatic techniques that allowed their description since the 1970s [12, 13]. In fact, because the iso-enzymatic technique required the near total destruction of the specimen and because the two species are sympatric in the type locality of *Ae. coluzzii*, no holotype of this species was designated by the species authors. Brengues et al. [2] developed an original multiplex polymerase chain reaction (PCR) to differentiate the two sibling species. They had the aim of depositing a neotype using this technique, but designation attempts failed and no neotype was finally deposited. The curator (third author of the present article) confirms that no specimen was deposited in the collection ARthropods of Medical Interest (ARIM) in Montpellier, contrary to what was claimed in [2]. In a nutshell, there is neither holotype nor neotype for the species *Ae. coluzzii*. Because we consider that a name-bearing type is necessary to define the nominal taxon objectively and in agreement with ICZN article 75, we here report the deposit of the fully undamaged neotype using an innovative method of identification through molecular analysis performed on the pupal exuvia of an adult male collected in the type-locality.

## Materials and methods

Our study approach on molecular identification of adult mosquito from pupal exuvia was based primarily on two published studies [3, 9]. We also used [18], although this work succeeded in amplifying DNA from a single mosquito leg but not from the pupal exuvia. However, a number of changes described below have been made.

### Mosquito collection

A field collection was performed on 24 February 2021 in Camargue at the type-locality of *Ae. coluzzii*, Salin-de-Giraud, Bouches-du-Rhône, Provence-Alpes-Côte d’Azur, France. The prospected site was a single water body, close to sea-shore along the “roubine” channel, located at the entrance of the track leading to the Mas du Clos d’Argent, 43°22′21,4″N, 4°48′31,7″E, about 1 m of elevation above sea level, with high salinity (36.9 g/L) close to that of sea water, pH 7.6, with salt vegetation; all stages of larvae and many pupae were collected at the same site, and adults were collected resting in vegetation (not aggressive at collection time, in the morning).

Four-instar larvae and pupae were collected and transported to Montpellier. Those morphologically identified as of the Detritus Complex were individually reared up to the adult stage. Adults were gingerly mounted on minute insect pins for the preservation of morphology to be studied through further microphotographs and scale counting (see below), and exuviae were placed in ethanol 95° and stored at −20 °C; each adult linked to its pupal exuvia received the same unique label.

### DNA extraction

Genomic DNA from the exuviae were individually extracted adapting the protocol described in [10]. Each exuviae was first washed in 400 μL of DNase-free water (Hyclone) for 2 min. Then it was transferred to a new 1.5 mL tube (Eppendorf) with 200 μL of 2% cetyltrimethylammonium bromide (CTAB) and ground with a micro-pestle. The solution was incubated at 65 °C for 10 min. Then 200 μL of chloroform were added, mixed by inversion and centrifuged 5 min at 12,000 rpm, at 18 °C. The top aqueous solution was transferred into a new 1.5 mL tube (Eppendorf), added 400 μL of cold (−20 °C) isopropanol, and placed in a freezer (−20 °C) to accentuate the precipitation. The solution was vortexed for 5 s and then centrifuged for 15 min at 15,000 rpm, at 18 °C. The supernatant was removed and the pellet washed with 200 μL of ethanol 75%. The solution was centrifuged again for 5 min at 12,000 rpm, at 18 °C. Again, the supernatant was removed and the pellet dried for 10 min in a vacuum at 40 °C. Finally, the DNA was resuspended in 20 μL of DNase-free water (Hyclone) in individual tubes overnight at room temperature for complete resuspension.

### DNA amplification

Primers used for ITS2 amplification were the same as those used in [1] also used in [2] (ITS2A: 5′–TGTGAACTGCAGGACACAT–3′/ITS2B: 5′–TATGCTTAAATTCAGGGGGT–3′). All PCR reactions were performed in 25 μL final volume, including 2 μL of exuviae DNA, 1× buffer (Eurogentec), 1.5 mM of MgCl_2_ (Eurogentec), 0.2 mM of dNTPmix (5 mM) (Eurogentec), 10 pmol of each primer and 1 Unit of Diamond Taq DNA polymerase (5 U/μL) (Eurogentec). The PCR amplifications were carried out in a Vapo Protect Thermocycler^®^ (Eppendorf). Cycling conditions were an initial denaturation at 94 °C for 2 min, followed by 40 cycles of 30 s denaturation at 94 °C, 30 s annealing at 52 °C and 45 s extension at 72 °C, and a final extension step of 72 °C for 10 min. After amplification, 10 μL of final PCR product was deposit onto a 2% agarose gel containing 8 μL of EmeralDye ClearLine^®^. The individuals exhibiting a visible and unique band on the agarose gel were bidirectionally sequenced by Eurofins genomics.

### DNA sequencing and phylogenetic analysis

The fragments resulting from sequencing the ITS2 region were manually corrected using Genious Prime (Biomatters Ltd.). The consensus sequences were aligned with known *Ae. coluzzii* and *Ae. detritus* sequences obtained from GenBank. For further phylogenetic analysis, we retained only the full sequences between 360 and 366 bp. We then inferred the phylogenetic tree using a maximum-likelihood algorithm via PhyML 3.0 [4] with an automatic model selection SMS (Smart Model Selection) [7]. The tree was visualized with iTOL v.5 [8].

### Morphological observations

High quality microphotographs of the neotype were taken with a Leica Z16ApoA stereomicroscope equipped with a DMC5400 camera. All pictures were made using a focus stacking technique (multiple images taken at different focus in order to extend the depth of field) within LAS X software from Leica. All pictures were then processed in Adobe Photoshop 2021 to correct and adjust various parameters such as exposure, white balance and light curve.

Count of pale scales of the abdominal terga was performed on the dark part of each tergum, (i.e. excluding the basal pale band and the apical pale band) for tergum I to VI (I-Te to VI-Te) to invest if this count could be used for morphological distinction between both species. Results are presented only for IV-Te and V-Te (see below).

## Results and discussion

In total, 77 adults of *Ae. detritus* s.l. (51 males and 26 females) emerged after a maximum of 3 weeks. From these, the DNA of 34 exuvia (30 males and 4 females) was extracted. We sequenced 21 individuals and identified 20 to species level. The success rate of 59% (=20/34) may appear to be poor performance but is in line with the minimum amount of DNA in the exuvia, mainly consisting of non-cellular epicuticle and exocuticle. For phylogenetic analysis, we kept 11 sequences with at least 363 bp.

### Taxonomic summary

*Aedes* (*Ochlerotatus*) *coluzzii* Rioux, Guilvard & Pasteur, 1998 (Insecta: Diptera: Culicidae)

Type locality: Salin-de-Giraud, Bouches-du-Rhône, France

Neotype: male specimen, label G20

Neotype deposition: ARIM (ARthropodes d’Intérêt Médical) collection at IRD-Délégation régionale Occitanie, 911 avenue Agropolis, Montpellier, France

Molecular information: ITS2 sequence of the neotype, GenBank accession number: OL471041.

### Species identification and neotype designation

The breeding site contained the 2 species of the complex in similar proportions: 11 *Ae. coluzzii* (10 males and 1 female) and 9 *Ae. detritus* (9 males). Clearly, the two species are able to grow in real sympatry (time and place) in an aquatic breeding site with a very high salt content (>36 g/L), a rate equivalent to that of sea water.

We designated as neotype the specimen with code G20, a male in good state of conservation with a complete ITS2 sequence (365 bp fully reliable for each nucleic acid). The other specimens in the collection are labelled with the codes G21, G27, P6, P8, V16, V19 and V30 for *Ae. coluzzii*, and G23, G26, P5, V2, V3, V4, V7, V18, V24 for *Ae. detritus*.

The ITS2 sequence of the neotype was deposited in GenBank (accession number OL471041).

**Figure 1:**
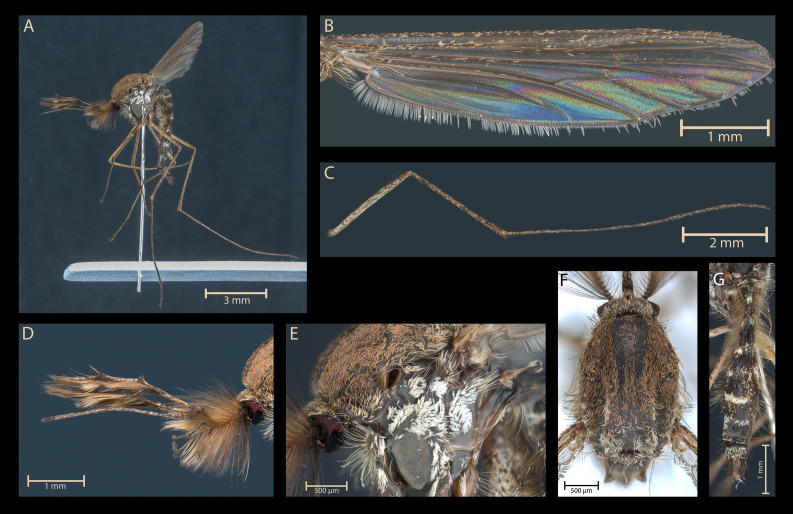
Photographs of the neotype of *Aedes* (*Ochlerotatus*) *coluzzii* male (specimen code G20): (A) general view, (B) right wing, upper view, (C) hind left leg, lateral left view, (D) anterior part, lateral view, (E) thorax, lateral left view, (F) scutum, (G) abdominal terga.

A plate of high-quality microphotographs showing external characters of morphological interest is proposed for the neotype (Fig. 1).

To our knowledge, this is the first time the type of a mosquito species is deposited thanks to its molecular identification from the pupal exuvia. Of interest, this method may be used in the future with the aim of designating various fully undamaged types, not only neotype, from specimens belonging to species complex.

### Morphological investigations

Roubaud and Treillard [15, 16] observed that the relative abundance of the pale scale coating on the dark part of the abdominal terga of *Ae. detritus* s.l. from the Camargue and the nearby Crau plain could vary greatly, from a dense seedling of pale scale to an almost total absence. They hypothesized that these differences were inferred by environmental characteristics of breeding sites: pale form with many pale scales in coastal saltwater “biotypes” *vs.* dark form with few pale scales in inland freshwater. In further experimental studies, they demonstrated that this assumption was unfounded, the number of the pale scales manifesting as a heritable attribute, independent of the salinity of the water in which the aquatic stages were reared [16].

**Figure 2:**
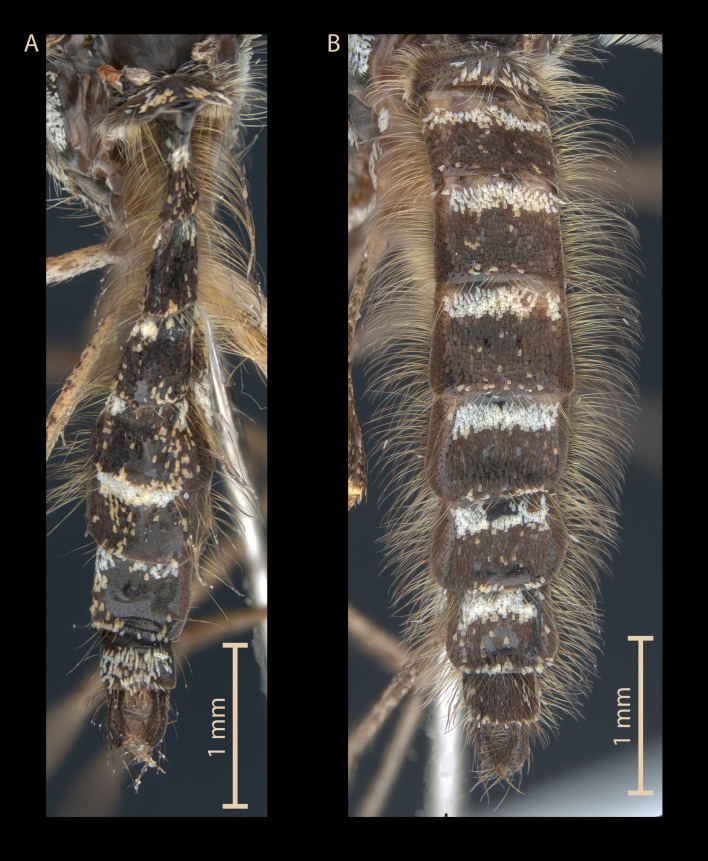
Morphological comparison of the abdominal terga of two male *Aedes coluzzii*: (A) the neotype specimen (specimen code G20) with a relatively high number of pale (white or yellowish) scales, (B) a specimen (code V19) with a relatively low number of pale scales.

**Figure 3:**
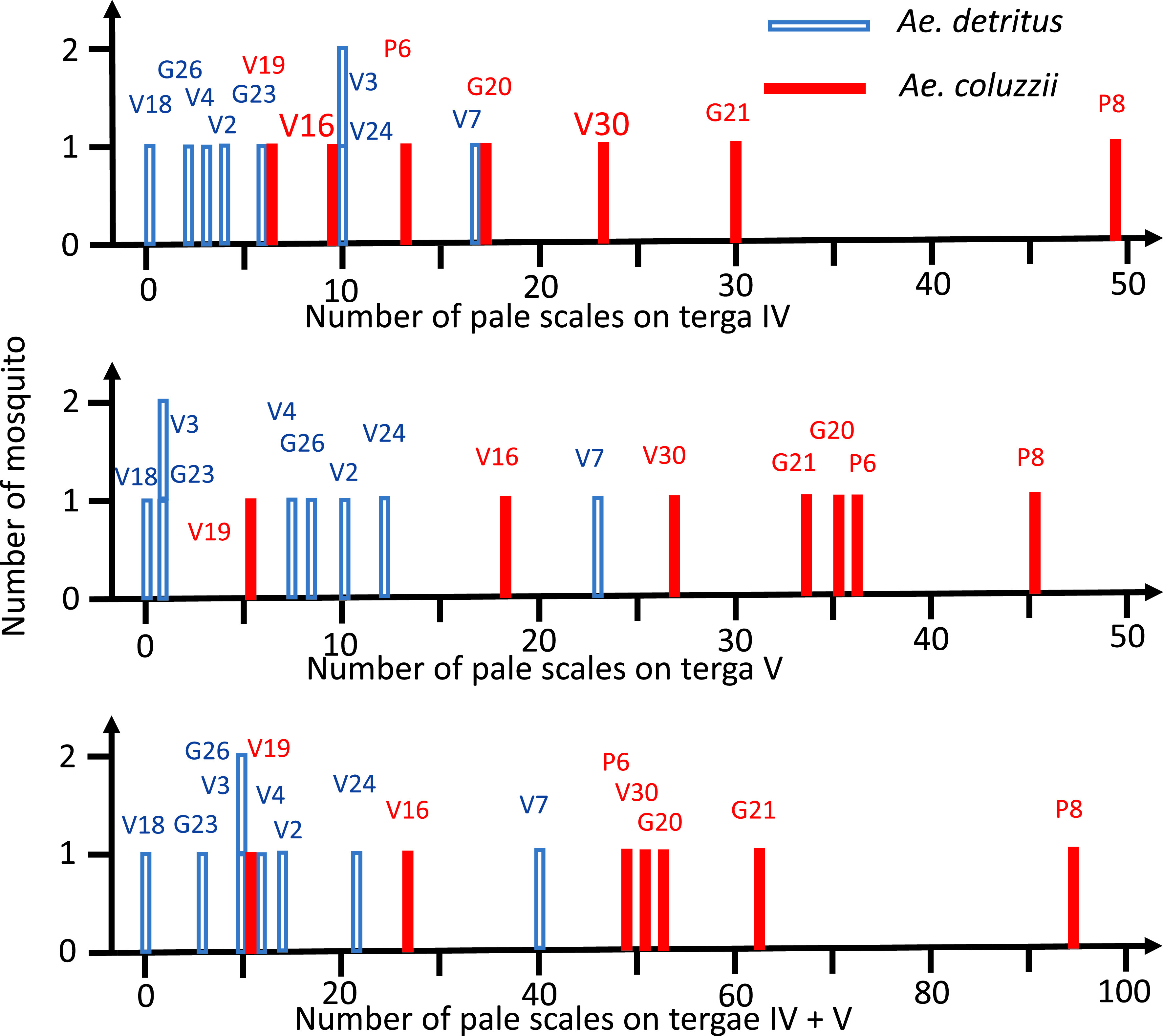
Distribution of the number of pale (white or yellowish) scales on the dark part of the abdominal tergae IV and V, with indication of the specimen code, for 8 male *Aedes* (*Ochlerotatus*) *detritus* and 7 male *Ae.* (*Och.*) *coluzzii*, collected from a single breeding site.

With this in mind, we tested whether the variation in scales counting could result from the existence of several species. We counted the pale scales (whitish or yellowish) on tergum segments IV and V on our specimens from the rearing of pre-imaginal forms collected in the same larval site and identified from a molecular point of view (7 males and 1 female *Ae. coluzzii*, 8 males *Ae. detritus*). This number of pale scales varies (Fig. 2). Importantly, male specimens of *Ae. coluzzii* exhibit more pale scales than *Ae. detritus* (Fig. 3). For tergum IV (IV-Te), the mean number of scales was 21.0 for *Ae. coluzzii vs*. 6.5 for *Ae. detritus* (*p* = 0.024 by non-parametric Wilcoxon-Mann Whitney two-sided test); for V-Te, 28.4 *vs*. 7.7, respectively (*p* = 0.013). But the distributions overlap partially (for IV-Te, the range is 6–49 pale scales for *Ae. coluzzii vs*. 0–17 for *Ae. detritus*; and for V-Te, 5–45 *vs*. 0–23, respectively) and therefore the count of pale scales cannot be retained as totally reliable for species diagnostic.

### Phylogenetic analysis

We generated 11 partial (between 363 and 369 bp) ITS2 sequences in this study (10 males *Ae. coluzzii* and 1 male *Ae. detritus*). They were exactly identical to those found by Brengues and colleagues [2]. The *Ae. detritus* specimen (code V3) corresponds to the previously called haplotype 2 (h2), while the other specimens represent the unique h3 corresponding to *Ae coluzzii* [2]. No hybrids were observed, in particular in the portions of the sequence that are diagnostic of species or haplotypes.

Other studies reported h1 in Greece (GenBank MG232616 [17]) and h2 in Tunisia (MN947506 and MN947508). h4, diagnostic of *Ae. detritus* and up to now not observed in France, has been found in Tunisia (KJ661031, MN947509 and MN947510).

**Figure 4:**
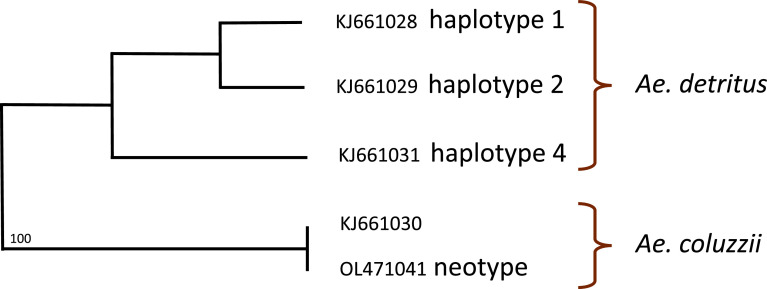
Unrooted maximum-likelihood phylogenetic tree based on rDNA ITS2 sequence polymorphism with indications of the GenBank accession numbers. Only significant values of bootstrap (obtained after 1000 replications) are mentioned.

A phylogenetic tree based on rDNA ITS2 sequence polymorphism is proposed in Figure 4. *Aedes coluzzii* appears to be isolated from its sibling species *Ae. detritus*. This tree was generated with exactly the same ITS2 sequences as in Figure 2 of Brengues and colleagues [2]. However, the two trees seem to be different, potentially due to the basal position of the taxa. Once checks were performed, it appeared that the tree published in [2] must be corrected in inversing the two indications “*Oc. coluzzii*” and “*Oc. detritus* h4”, and in this condition, the two trees are identical. We propose to keep this new tree for future phylogenetic comparisons.
